# IL-15Rα-Independent IL-15 Signaling in Non-NK Cell-Derived IFNγ Driven Control of *Listeria monocytogenes*

**DOI:** 10.3389/fimmu.2021.793918

**Published:** 2021-12-10

**Authors:** Madhuparna Nandi, Mitterrand Muamba Moyo, Sakina Orkhis, Jeanne Masunga Faida Mobulakani, Marc-André Limoges, Fjolla Rexhepi, Marian Mayhue, Anny Armas Cayarga, Gisela Cofino Marrero, Subburaj Ilangumaran, Alfredo Menendez, Sheela Ramanathan

**Affiliations:** ^1^ Department of Immunology and Cell Biology, Université de Sherbrooke, Sherbrooke, QC, Canada; ^2^ Department of Microbiology and Infectious Diseases, Université de Sherbrooke, Sherbrooke, QC, Canada; ^3^ Centre de Recherche du Centre Hospitalier Universitaire de Sherbrooke (CRCHUS), Sherbrooke, QC, Canada

**Keywords:** IL-15, *Listeria*, IFN-gamma, IL-15 Receptor alpha, infection, spleen, mice

## Abstract

Interleukin-15, produced by hematopoietic and parenchymal cells, maintains immune cell homeostasis and facilitates activation of lymphoid and myeloid cell subsets. IL-15 interacts with the ligand-binding receptor chain IL-15Rα during biosynthesis, and the IL-15:IL-15Rα complex is trans-presented to responder cells that express the IL-2/15Rβγ_c_ complex to initiate signaling. IL-15-deficient and IL-15Rα-deficient mice display similar alterations in immune cell subsets. Thus, the trimeric IL-15Rαβγ_c_ complex is considered the functional IL-15 receptor. However, studies on the pathogenic role of IL-15 in inflammatory and autoimmune diseases indicate that IL-15 can signal independently of IL-15Rα *via* the IL-15Rβγ_c_ dimer. Here, we compared the ability of mice lacking IL-15 (no signaling) or IL-15Rα (partial/distinct signaling) to control *Listeria monocytogenes* infection. We show that IL-15-deficient mice succumb to infection whereas IL-15Rα-deficient mice clear the pathogen as efficiently as wildtype mice. IL-15-deficient macrophages did not show any defect in bacterial uptake or iNOS expression *in vitro*. *In vivo*, IL-15 deficiency impaired the accumulation of inflammatory monocytes in infected spleens without affecting chemokine and pro-inflammatory cytokine production. The inability of IL-15-deficient mice to clear *L. monocytogenes* results from impaired early IFNγ production, which was not affected in IL-15Rα-deficient mice. Administration of IFNγ partially enabled IL-15-deficient mice to control the infection. Bone marrow chimeras revealed that IL-15 needed for early bacterial control can originate from both hematopoietic and non-hematopoietic cells. Overall, our findings indicate that IL-15-dependent IL-15Rα-independent signaling *via* the IL-15Rβγ_c_ dimeric complex is necessary and sufficient for the induction of IFNγ from sources other than NK/NKT cells to control bacterial pathogens.

## Introduction

Interleukin-15 (IL-15) is a multi-faceted cytokine that facilitates the activation of myeloid and lymphoid components of the innate immune response to pathogens (1–3). IL-15 is expressed by activated macrophages and dendritic cells (DC) as well as stromal cells in the bone marrow and thymus and intestinal epithelial cells (4). Even though IL-15 transcripts are detected in different organs such as skeletal muscle, intestine and kidney at steady state (5), protein expression is not detectable by conventional methods (4). The IL-15 receptor (IL-15R) is a trimeric complex composed of the ligand-binding IL-15Rα chain, the β chain shared by IL-2 and IL-15 (IL-2/15Rβ) and the common γ_c_ chain (1). Mice lacking either IL-15 or IL-15Rα display reduced numbers of NK and NKT cells, memory CD8^+^ T lymphocytes and intestinal epithelial lymphocytes (IEL), indicating that IL-15Rα is crucial for their IL-15-dependent homeostasis (6, 7). Transgenic expression of human IL-15 increases NK and memory CD8^+^ T cells but not monocytes or B cells (2, 8, 9). Mechanistic studies revealed that the biological activities of IL-15 on lymphoid cells are mediated by IL-15 bound to IL-15Rα during biosynthesis and ‘trans-presentation’ of the IL-15:IL-15Rα (IL15:15Rα) complex to responder cells expressing the IL-2/15Rβγ_c_ heterodimeric receptor complex, explaining the similarity in the phenotype of *Il15^-/-^* and *Il15ra^-/-^* mice (6, 7, 10).

Studying the mechanisms of IL-15 signaling has remained challenging due to post-transcriptional and post-translational regulation of IL-15 expression and the requirement for IL-15 trans-presentation (11, 12). In producer cells, IL-15 protein is synthesized with either a short or a long signal peptide (SSP and LSP, respectively). Whereas the SSP-IL-15 is not released from cells, LSP-IL-15 is inefficiently secreted (13). As transgenic mice expressing LSP-IL-15 mounted a robust immune response following *Salmonella* infection, whereas mice expressing the non-secreted SSP-IL-15 did not, it was postulated that the over-expressed SSP-IL-15 isoform may compete with endogenous IL-15 for binding to IL-15Rα and inhibit IL-15 signaling (14). While soluble IL-15 can promote activation of NK and memory CD8^+^ subsets (7), such high concentrations are unlikely to occur under physiological settings. Free IL-15 can bind the IL-2/15Rβγ_c_ receptor complex at nanomolar concentration, however, IL-15Rα facilitates ligand binding at picomolar concentrations (15, 16). IL-15 trans-presented by hematopoietic as well as non-hematopoietic cells is the primary mechanism by which IL-15 activates lymphocytes (17). Tissue specific ablation of IL-15Rα has revealed specific, but distinct patterns of requirement for the source of the trans-presented IL-15 (18, 19). We have shown IL-15 trans-presentation by hepatocytes and macrophages is required for the maintenance of NK and NKT cells in the liver (20).

A few studies comparing the immune responses of *Il15^-/-^* and *Il15ra^-/-^* mice indicated that IL-15 trans-presentation is not the only mechanism of IL-15 signaling and that it can also occur independently of IL-15Rα, although the underlying mechanisms are not yet fully understood. We have shown that the pathogenic potential of IL-15 in autoimmune type 1 diabetes (T1D) does not require IL-15Rα (21, 22). In the dextran sulfate sodium-azoxymethane (DSS-AOM)-induced colitis-associated carcinogenesis model, *Il15^-/-^* mice but not *Il15ra^-/-^* mice displayed higher incidence of colorectal cancer and reduced survival (23). These differences, which cannot be explained solely by the lack of NK cells that are similarly affected in IL-15 or IL-15Rα deficient mice (6, 24), indicated that IL-15 can mediate certain aspects of the immune responses independently of IL-15Rα. Moreover, an enlarged compartment of IL-17 producing γδ T cells has been observed in *Il15ra^-/-^* but not in *Il15^-/-^* mice, suggesting IL-15Rα-independent IL-15-mediated regulation (25).

Using IL-15 deficient mice, several studies have established the requirement of IL-15 to efficiently control bacterial, viral and fungal infections (3, 26, 27) presumably through its functions on lymphoid subsets, which require trans-presentation. However, IL-15Rα-independent functions of IL-15 in antimicrobial defenses are poorly characterized. In this work, we compared the requirement of IL-15 and IL-15Rα in controlling *Listeria monocytogenes* infection and determined that IL-15 signaling independent of IL-15Rα is critical for the induction of IFNγ and bacterial clearance.

## Materials and Methods

### Mouse Strains

All the mice strains used in this study were bred in our animal facility to avoid major differences in microbiome and environmental influences. Wild type C57Bl/6N (WT) mice, *Il15^-/-^*, *Il15ra^-/-^* and *Ifng^-/-^* mice in the C57Bl/6N background have been described previously (22, 28–30). Mice were housed in micro-isolated sterile cages under specific pathogen-free conditions. All experimental protocols on mice were approved by Université de Sherbrooke Ethics Committee for Animal Care and Use in accordance with guidelines established by the Canadian Council on Animal Care (protocol # 376-2014-2019; 2019-2023-2280).

### Bacterial Strains, Infections and Pre/Post Infection Treatments

Frozen glycerol stock of *L. monocytogenes* strain 10403s and *L. monocytogenes* expressing green fluorescent protein (GFP) were obtained from Dr. Portnoy (Department of Molecular and Cell Biology, University of California, Berkeley, CA). Bacteria were cultured in BHI (Blood-heart infusion) medium (VWR, Canada). Mice were injected intravenously in the tail vein with 5 000 to 10 000 colony forming units (CFU) of bacteria for low-dose infections and with 100 000 CFU for high-dose infections. Liver and spleen were collected, and tissue lysates were prepared in 1 mL of PBS from weighed pieces using a tissue homogenizer bead mill (MM 400; Retsch, Hann, Germany). Bacterial burden in tissues was determined by plating serial dilutions of the homogenates on BHI agar plates. Indicated groups of mice received intraperitoneal injections of IFNγ (1 x10^6^ units, R&D Systems, on day 0 and day 1), anti-NK1.1 (PK136; 200ug/mouse/injection at day -1 and +1) or anti-IFNγ (H22; 200ug/mouse/injection at day -1 and +1) antibodies (all from BioXCell, USA) relative to the infection day 0.

### Bone Marrow-Derived Macrophages (BMDM)

BMDMs were generated following standard methods (31) with some modifications. Briefly, bone marrow cells collected from femurs of the indicated mice were seeded at a density of 5 ×10^5^ cells per well in 24-well plates and at 10^6^ cells per well in 12-well plates in RPMI medium containing 15% fetal bovine serum (FBS) the presence of 50 ng/mL of macrophage colony stimulating factor (M-CSF, Peprotech, USA) for 5 days. M-CSF was supplemented on Day 4. To generate BMDMs grown in granulocyte- macrophage colony stimulating factor (GM-CSF, Peprotech, USA), the cytokine was added to the above cultures at a concentration of 50 ng/mL on day 6 and the cells were used on day 7.

### Infection of BMDM

An overnight culture of *L. monocytogenes*, which contains approximately 2 x 10^9^ CFU/mL, was washed and resuspended in the same volume of PBS. Twenty five μL of washed bacteria were opsonized by mixing with 20 μL of pre-immune mouse serum and 55 uL RPMI (without serum or antibiotics) for 30 min at 25°C. The inoculum was prepared by diluting the opsonized bacteria in RPMI (100 μL of opsonized bacterial suspension in 3ml RPMI) to provide a multiplicity of infection (MOI) of ~25. The MOI was determined for every experiment by plating the inoculum at 10-fold serial dilutions (10^-3^, 10^- 4^, 10^-5^) on BHI agar plates. These plates were incubated at 37°C from 24 hours, and colonies counted to determine the CFU.

Prior to infection, medium was removed from the BMDM culture plates by gentle suction and the wells were washed once with warm RPMI medium without FCS or antibiotics. Opsonized bacteria (400 or 800 ul/well) were added to BMDMs and the infected cells were incubated at 37°C and 5% CO_2_ for 30 minutes followed by washing the wells with 1 mL of PBS containing Ca^2+^ and Mg^2+^. The wells were replenished with RPMI containing 10% FBS and 20 μg/mL gentamicin to kill the extracellular bacteria. After 4 hours, cells were recovered from the plates for flow cytometry analysis. Another batch of culture was washed with PBS and lysed in 200 μL of lysis buffer containing 1% triton X100/0.1% SDS for counting the intracellular bacteria. Numbers of viable intracellular *L. monocytogenes* were determined by performing 10- fold serial dilutions (10^-1,^ 10^-2^, 10^-3^) and plating on BHI agar plates for 14–16 hr at 37°C for CFU quantification.

### Preparation of Splenocyte Suspensions

Splenocytes were isolated and dissociated in PBS containing 1% FBS and penicillin and streptomycin. The cells were labeled with fluorochrome-conjugated antibodies for flowcytometry as described (22, 32). The list of antibodies used is given in **Supplementary Table S1**. Cells were fixed after surface staining and permeabilized for 30 minutes using a wash buffer (BD Biosciences) following manufacturer’s protocols for intracellular staining of iNOS. Cells were analyzed on a Cytoflex 30 flow cytometer (Beckman Coulter, Brea, CA, USA) and data analyzed using the FlowJo software (BD Biosciences).

### Data Analysis

Graphpad Prism software (Version 9) was used for data analysis and to generate illustrations. The data are shown as Mean + standard error of mean (SEM). One- or Two-way ANOVA with Tukey’s or Bonferroni’s *post-hoc* test was used as indicated in figure legends to compare datasets and to calculate statistical significance.

## Results

### IL-15 Is Crucial but IL-15Rα Is Dispensable for Clearing Low-Dose *L. monocytogenes* Infection

**Figure 1:**
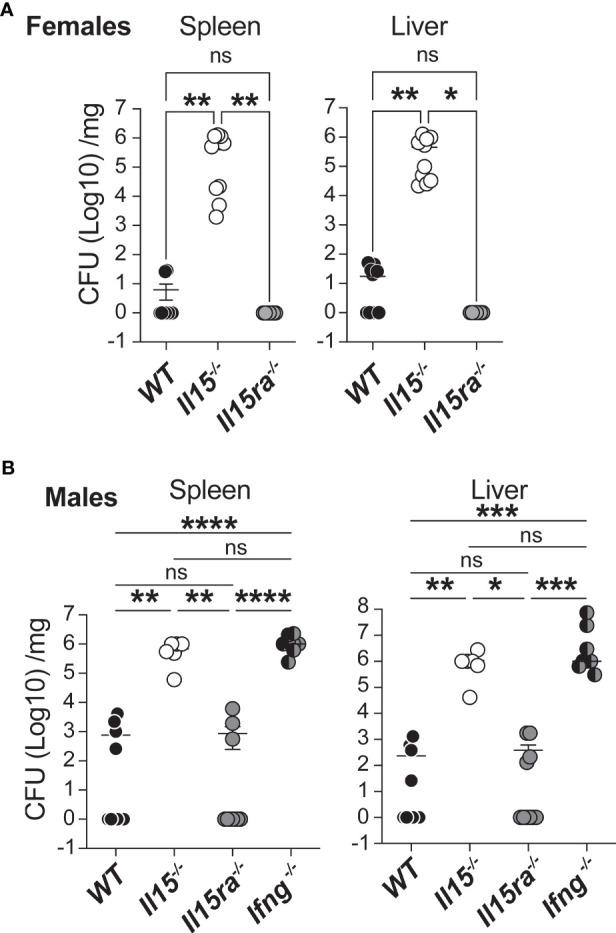
IL-15 is essential and IL-15Rα is dispensable for clearing systemic infection with low dose *L. monocytogenes*. **(A)** Female WT, *Il15-/-* and *Il15ra-/-* mice were infected i.v. with 5 x10^3^ CFU (colony forming units) of *L. monocytogenes.* The infected mice were sacrificed 5 days post infection (dpi) and the bacterial load in the spleen and liver was determined by plating on BHI-streptomycin plates. **(B)** Bacterial load in male WT, *Il15-/-*, *Il15ra-/-* and *Ifng-/-* mice infected i.v. with 5 x10^3^ CFU of *L. monocytogenes.* Bacterial count is expressed as CFU per mg of tissue. Results are shown as the Mean ± SEM of two independent experiments. n = 5 to 10 mice per group. Statistical significance between groups determined by one-way ANOVA followed by Tukey’s multiple comparisons test, is given as *p < 0.05, **p < 0.01, ***p < 0.001 and ****p < 0.0001. ns, not significant.

WT, *Il15-/-* and *Il15ra-/-* mice were infected with a low dose of 5 ×10^3^ CFU of *L. monocytogenes via* the intravenous route. At 5 days post infection (dpi), female WT mice were able to control the infection and showed reduced bacterial load in spleen and the liver, whereas female *Il15-/-* mice showed high bacterial counts in these tissues (**Figure 1A**). In contrast to *Il15-/-* mice, *Il15ra-/-* animals cleared *L. monocytogenes* from the spleen and the liver as efficiently as WT mice (**Figure 1A**). Male mice have been reported to better resist *L. monocytogenes* infection compared to female mice (33). However, at the infection dose used in our studies, we did not observe appreciable difference between male and female mice in their ability to clear *L. monocytogenes* (**Figure 1B**). Similar to females, WT and *Il15ra-/-* male mice efficiently cleared splenic and hepatic *L. monocytogenes* within 5 dpi, whereas *Il15-/-* mice did not. The bacterial load observed in *Il15-/-* male mice was comparable to that of *Ifng-/-* male mice, indicating that IL-15 is as important as IFNγ in controlling *L. monocytogenes* infection (**Figure 1B**). The dispensability of IL-15Rα but not IL-15 to clear low dose *L. monocytogenes* infection indicated that IL-15 signaling can occur independently of IL-15Rα and that this signaling plays a key role in the control of bacterial infections.

### IL-15 Deficiency Does Not Affect the Infection of BMDMs by *L. monocytogenes*

**Figure 2:**
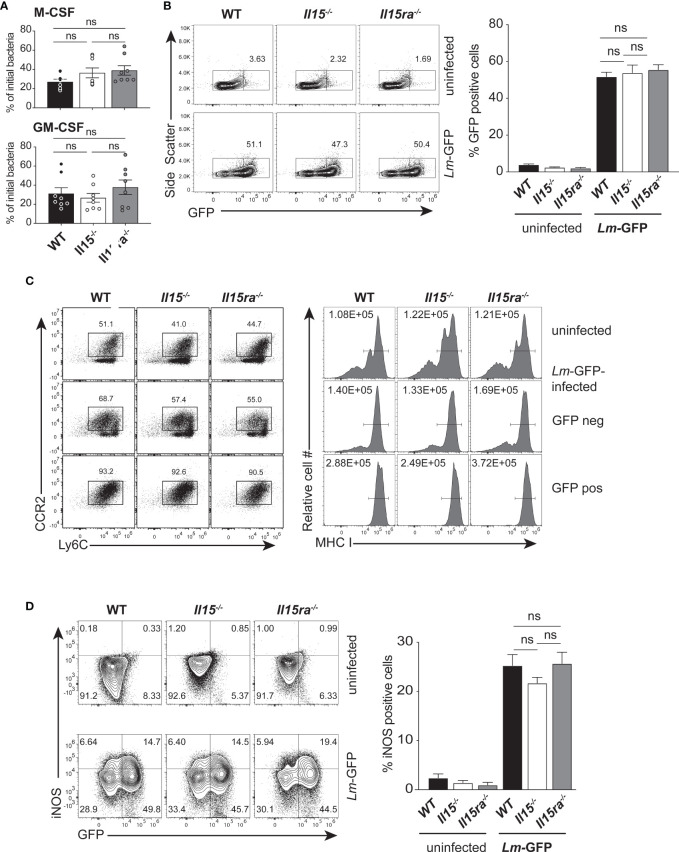
IL-15 deficient BMDMs are infected by *L. monocytogenes*. **(A)** BMDMs generated in the presence of M-CSF (upper panel) or treated additionally with GM-CSF for 1 day (lower panel) were infected with opsonized *L. monocytogenes* expressing GFP (*Lm*-GFP) at a MOI of 25. The cells were washed 30 minutes after infection and incubated in medium containing gentamycin for additional 4 hours before cell lysis. The number of intracellular bacteria were quantified by plating dilutions of cell lysates on BHI-agar and expressed as a fraction of the bacterial inoculum. **(B)** The proportion of CD11b+ cells that are positive for GFP 4 hours after infection was determined by flow cytometry. Representative flow cytometry data (left panel) and quantification of GFP+ cells (right panel) are shown. **(C)** The expression of Ly6C and CCR2 (left Panel) and MHC-I (right Panel) on uninfected controls and GFP+ and GFP-cells from the infected cultures are shown. Representative FACS data from one of the 3 experiments are shown. **(D)** BMDMs generated using M-CSF were infected with *Lm*-GFP as in **(A)** and labelled with CD11b and iNOS Ab for flow cytometry 4 h later. The dot plots (left panel) show iNOS expression in GFP+ and GFP- cells of infected and control cultures. The proportions of iNOS-positive cells among GFP+ cells are shown in the right panel **(A)** and Right panels in **(B, D)**. Results are shown as the mean ± SEM of 3 independent experiments carried out in triplicates. Statistical significance between groups, determined one-way ANOVA followed by Tukey’s multiple comparisons test, is given. ns, not significant.

To investigate whether the IL-15Rα-independent IL-15 signaling impacted bacterial uptake by phagocytic cells, we used bone marrow-derived macrophages (BMDM) established from WT, *Il15-/-* and *Il15ra-/-* mice. BMDMs cultured with M-CSF were infected at a MOI of 25 with *L. monocytogenes* expressing GFP (*Lm*-GFP) that were opsonized with non-immune mouse serum to facilitate complement-mediated uptake. Thirty min after infection, cells were washed to remove extracellular bacteria and incubated in medium containing gentamycin for additional 4 hours before detection of intracellular bacteria. The fraction of bacteria present within BMDMs was comparable between WT and *Il15-/-* cells 4 hours post-infection (hpi) (**Figure 2A**, upper panel). In BMDMs that were activated with GM-CSF for additional 24 hours (31), there was no significant difference among the three groups (**Figure 2A**, lower panel). Flow cytometry analysis revealed that the proportion of M-CSF-BMDMs harboring *Lm*-GFP was comparable between the three genotypes (**Figure 2B**). As BMDMs are diverse in their phenotype (34), it raised the possibility that lack of IL-15 signaling, in the presence or absence of IL-15Rα, might impact the frequencies of BMDM subsets with altered susceptibility to bacterial infection. To determine whether absence of IL-15 altered the infectivity of different subsets of BMDM, the control and infected cultures were labeled for CCR2, Ly6C and MHC class-I (MHC-I) and the GFP+ and GFP- subsets were analyzed. The GFP+ M-CSF-BMDMs from all three genotypes were predominantly Ly6C+CCR2+ (**Figure 2C** Left panel, bottom row) and upregulated MHC-I expression (**Figure 2C** Right Panel, bottom row) compared to the GFP- and uninfected cells (middle and top rows, respectively). Macrophages play an essential role in eliminating the *L. monocytogenes* infected cells by upregulating the expression of inducible NOS2 (iNOS, induced by inflammatory cytokines) for the production of reactive nitrogen species (RNS) (35). Expression of iNOS occurred in a significant proportion of GFP+ BMDM established from all three groups of mice and this proportion was comparable among the three genotypes (**Figure 2D**). These findings indicated that lack of either IL-15 or IL-15Rα had only a negligible impact on bacterial load in macrophages generated *in vitro* and their ability to produce iNOS.

### Infected IL-15-Deficient Mice Show Accumulation of Inflammatory Monocytes and Elevated Production of Many Chemokines and Pro-Inflammatory Cytokines in Spleen

**Figure 3:**
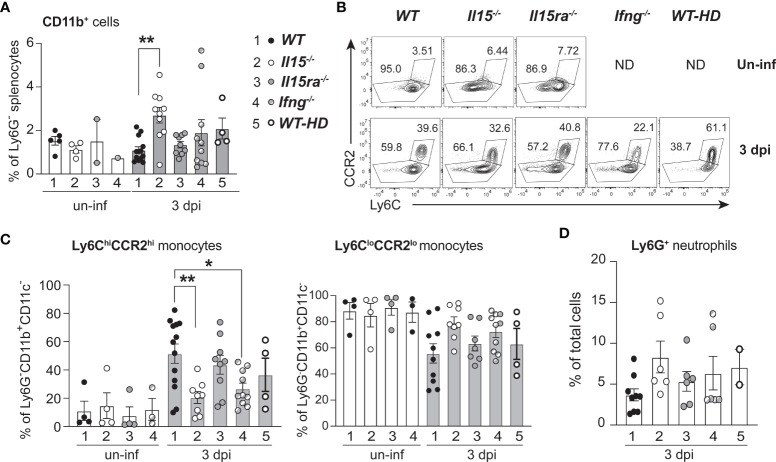
Lack of IL-15 but not IL-15Rα reduces the frequency of Ly6C+CCR2+ pro-inflammatory monocytes in the spleen following *L. monocytogenes* infection. **(A)** WT, *Il15-/-*, *Il15ra-/-* and *Ifng-/-* mice were infected i.v. with 5 x10^3^ CFU of *L. monocytogenes.* A cohort of WT mice were infected i.v. with higher dose (50-100 x10^3^ CFU; WT-HD) of *L. monocytogenes* to obtain higher bacterial load shown in **Supplementary Figure 1**. The infected mice were sacrificed at 3 dpi and the splenocytes were analyzed by flow cytometry. The frequency of CD11b+ cells is shown after gating out neutrophils (based on Ly6G positivity) and CD11c+ cells. **(B)** Representative density plots of Ly6C and CCR2 expression in CD11b+ cells. **(C)** Frequency of Ly6C+CCR2+ inflammatory monocytes (left panel) and Ly6CloCCR2lo monocytes (right panel) within the Ly6G-CD11b+CD11c- population. **(D)** Frequency of Ly6G+ neutrophils. Results are shown as the mean ± SEM values from n = 5 to 10 mice per group from of 2-4 independent experiments. Statistics: one-way ANOVA followed by Tukey’s multiple comparisons test. *p < 0.05, **p < 0.01.

Next, we determined how IL-15 or IL-15Rα deficiency impacted monocytes/macrophage phenotype *in vivo* in the spleens of *L. monocytogenes*-infected WT, *Il15-/-* and *Il15ra-/-* mice. The frequency of CD11b+ monocytes was substantially increased in infected *Il15-/-* mice 3 dpi when compared to infected WT and *Il15ra-/-* mice (**Figure 3A**). However, the frequency of Ly6ChiCCR2hiCD11b+ inflammatory monocyte subset showed a moderate but significant reduction in *Il15-/-* mice (**Figures 3B, C**). The Ly6CloCCR2loCD11b+ monocyte subset showed a corresponding increase that was not statistically significant. To determine whether the accumulation of Ly6ChiCCR2hiCD11b+ pro-inflammatory monocyte subset in *Il15-/-* mice was due to the increased bacterial load that occurs in *Il15-/-* mice (**Figure 1A**), we used 2 additional groups of mice wherein higher splenic bacterial load can be achieved similar to the levels attained in *Il15-/-* mice. *Ifng-/-* mice are susceptible to low dose *L. monocytogenes* infection (36) and WT mice infected with a high dose of *L. monocytogenes* (1 x 10^5^ CFU) succumb to disease (37). Even though the bacterial load in *Ifng-/-* mice and WT mice infected with a high dose inoculum were comparable to *Il15-/-* mice (**Supplementary Figure S1**), they did not show an increase in the frequency of CD11b+ cells as in *Il15-/-* mice (**Figure 3A**). However, *Ifng-/-* mice showed an increase in the frequency of the Ly6ChiCCR2hiCD11b+ pro-inflammatory monocyte subset like *Il15-/-* mice (**Figures 3B, C**). On the other hand, all groups showed comparable neutrophils infiltration in the spleen, although *Il15-/-* mice showed a discernible increase (**Figure 3D**). These results suggest that the lack of IL-15 but not that of IL-15Rα impacts the accumulation of Ly6ChiCCR2hiCD11b+ pro-inflammatory monocyte subset in the infected tissue.

**Figure 4:**
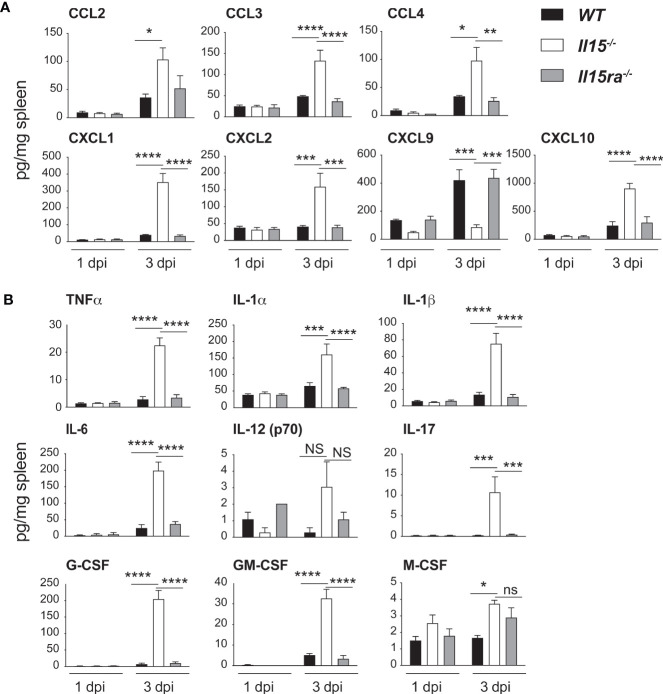
IL-15 deficient mice show increased chemokine and pro-inflammatory cytokine production but reduced CXCL9 expression following *L. monocytogenes* infection. WT, *Il15-/-* and *Il15ra-/-* mice were infected i.v. with 5 x10^3^ CFU of *L. monocytogenes.* The infected mice were sacrificed at 1 dpi or 3 dpi and spleen tissue lysates were analyzed by multiplex assays for the expression of **(A)** the indicated CCL and CXCL chemokines and **(B)** pro-inflammatory cytokines. Results are shown as the mean ± SEM of n = 4 mice per group from 2 independent experiments. Statistical significance between groups was evaluated by Two-way ANOVA followed by Bonferroni’s multiple comparisons test. *p < 0.05; **p < 0.01; ***p < 0.001; ****p < 0.0001; ns, not significant.

Chemokines play a key role in monocyte recruitment to inflammatory sites, of which CCL2 promotes the recruitment of Ly6C+CCR2+ inflammatory monocytes (38). To assess whether IL-15 deficiency compromises the induction of chemokines, we evaluated the chemokine levels in spleen lysates of control and *L. monocytogenes*-infected mice by multiplex assays. All infected mice groups showed an upregulation of several CCL (CCL2, CCL3, CCL4) and CXCL (CXCL1, CXCL2, CXCL9, CXCL10) chemokines (**Figure 4A**). However, the spleens of *Il15-/-* mice showed significantly elevated levels of most of the chemokines tested compared to WT and *Il15ra-/-* mice, except CXCL9, which showed an inverse trend with a significant reduction in *Il15-/-* mice (**Figure 4A**). CXCL9 is also known as monokine-induced by IFNγ (MIG) that recruits macrophages, NK, NKT and cytotoxic lymphocytes (39). The spleens of infected *Il15-/-* mice also showed high levels of many pro-inflammatory cytokines (TNFα, IL-1α, IL-1β, IL-6, IL-17) and granulocyte/macrophage growth factors (G-CSF, GM-CSF, M-CSF) (**Figure 4B**). These data indicated that the susceptibility to infection in *Il15-/-* mice is not due to the lack of production of most of the chemokines and cytokines associated with innate immune responses immediately after infection.

**Figure 5:**
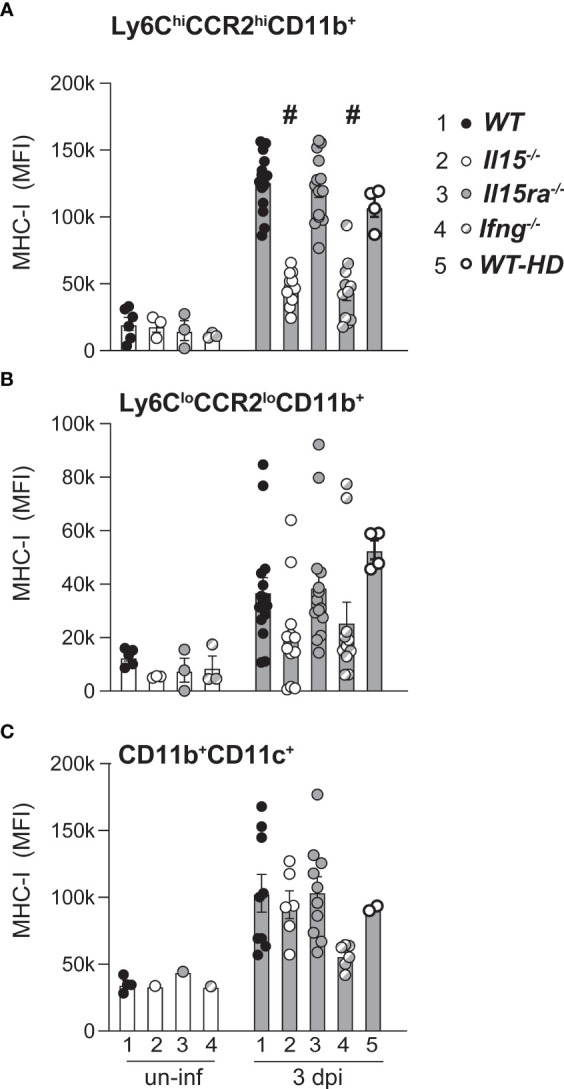
Attenuated MHC Class-I induction in inflammatory monocytes of *Il15-/-* mice following *L. monocytogenes* infection. Mean fluorescence intensity (MFI) of MHC class-I expression in Ly6C+CCR2+ inflammatory monocytes **(A)**, Ly6CloCCR2lo monocytes **(B)** and CD11b+CD11c+ monocytes **(C)** at 3 dpi. Results are shown as the mean ± SEM of n = 5 to 10 mice per group from 2-4 independent experiments. Statistical significance between groups, one-way ANOVA followed by Tukey’s multiple comparisons test. # p < 0.0001 when compared to groups 1, 3 and 5. Each uninfected group contained 3-4 mice.

To further characterize the response of monocytes/macrophage of *L. monocytogenes*-infected WT, *Il15-/-* and *Il15ra-/-* mice, we assessed the induction of MHC-I, using *Ifng-/-* mice and WT mice that were infected with high dose *L. monocytogenes* as controls to mimic the high bacterial load observed in *Il15-/-* mice. The basal expression of MHC-I on Ly6ChiCCR2hiCD11b+, Ly6CloCCR2loCD11b+ monocytes and CD11b+CD11c+ monocyte subsets was comparable in uninfected mice from the four genotypes, and *L. monocytogenes* infection induced MHC-I upregulation in all these monocyte subsets (**Figure 5A**). However, MHC-I expression in Ly6ChiCCR2hiCD11b+ monocytes was significantly lower in *Il15-/-* and *Ifng-/-* mice at 3 dpi when compared to WT and *Il15ra-/-* mice (**Figure 5A**). The attenuation of MHC-I upregulation in *Il15-/-* and *Ifng-/-* mice was also observed in Ly6CloCCR2loCD11b+ but did not show statistical significance (**Figure 5B**). On the other hand, MHC class I expression in the CD11b+CD11c+ monocyte subsets was comparable between the groups (**Figure 5C**). The impaired MHC-I upregulation in Ly6ChiCCR2hiCD11b+ monocytes of *Il15-/-* and *Ifng-/-* mice could not be attributed to the high bacterial load, as it was not affected in WT-HD mice with high bacterial load achieved by high dose infection (**Figure 5A**). Moreover, MHC-II expression on the inflammatory monocyte subset was comparable between the mice from different genotypes (**Supplementary Figure S2**). These findings revealed similarly impaired macrophage responses in *Il15-/-* and *Ifng-/-* mice that did not occur in *Il15ra-/-* mice.

### Defective Early IFNγ Production in *L. monocytogenes* Infected *Il15-/-* but Not *Il15ra-/-* Mice

**Figure 6:**
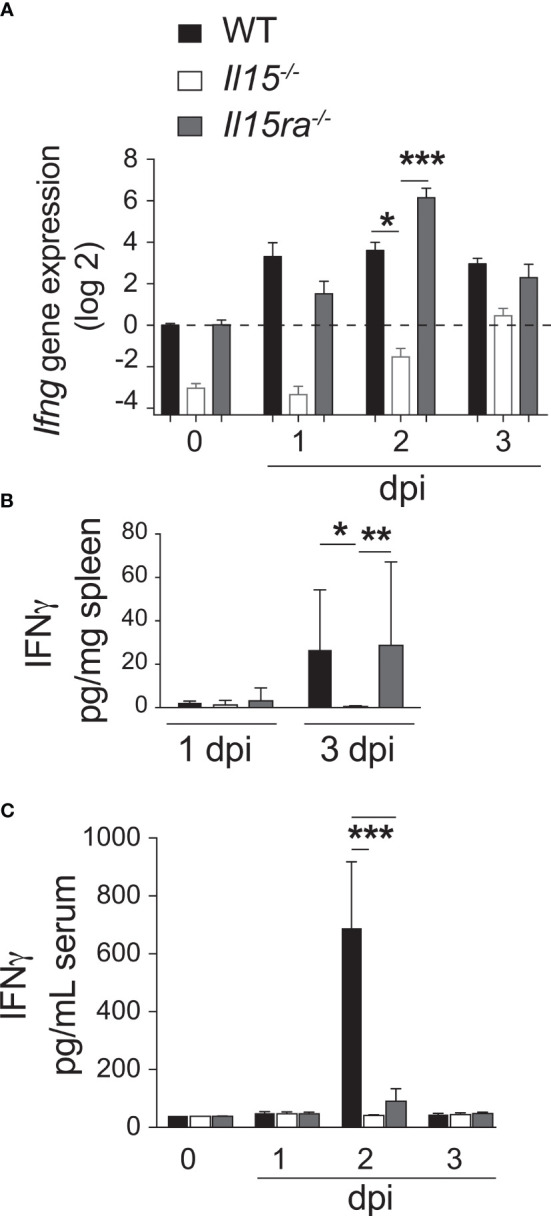
Lack of IL-15 but not IL-15Rα impairs early IFNγ production after *L. monocytogenes* infection. **(A)** WT, *Il15-/-* and *Il15ra-/-* mice were infected i.v. with 5 x10^3^ CFU of *L. monocytogenes.* The infected mice were sacrificed at 1, 2 or 3 dpi. **(A)** *Ifng* gene expression (fold induction over WT uninfected controls) was determined in the spleen by RT-qPCR. IFNγ protein concentration in spleen lysates **(B)** and sera **(C)** from control and infected mice by ELISA. Results are shown as the mean ± SEM for 4 mice per group. Statistical significance between groups, two-way ANOVA followed by Bonferroni’s multiple comparisons test, is given as *p < 0.05, **p < 0.01, ***p < 0.001.

The high bacterial load and reduced numbers of Ly6ChiCCR2hiCD11b+ pro-inflammatory monocytes in *Il15-/-* and *Ifng-/-* mice but not in *Il15ra-/-* mice (**Figure 3C**), and the impaired expression of the IFNγ-inducible CXCL9 in *Il15-/-* mice (**Figure 4A**) suggested defective IFNγ responses in the absence of IL-15 but not that of IL-15Rα in the infected mice. Ohteki et al. have reported that heat-killed *Propionibacterium acnes* (*P. acnes*) failed to induce IL-12, IFNγ, CCL2 and CCL3 in the livers of *Il15-/-* mice (40). Similarly, IL-15 deficient mice failed to produce IL-12 during *L. monocytogenes* infection (41). Low dose *L. monocytogenes* did not impair the production of IL-12p70, CCL2 or CCL3 in *Il15-/-* mice (**Figure 4A**). On the other hand, we observed that IL-15 deficiency but not IL-15Rα deficiency markedly reduced IFNγ expression. *Ifng* gene expression was induced as early as 1 dpi in the spleens of WT and *Il15ra-/-* infected mice and peaked around 2 dpi (**Figure 6A**). However, at these early time points, *Ifng* expression was very low in the spleen of *L. monocytogenes* infected *Il15-/-* mice. Even though *Ifng* expression in these mice gradually increased at 3 dpi, this level was comparable only to that of uninfected WT and *Il15ra-/-* mice, which was not sufficient to confer protection, as all infected *Il15-/-* mice succumbed by 5 dpi. IFNγ protein was detected by 3 dpi in the spleens of WT and *Il15ra-/-* but was undetectable in *Il15-/-* mice (**Figure 6B**).

Notably, IFNγ was detected in the sera of infected WT mice and at very low levels in *Il15ra-/-* mice but not in *Il15-/-* mice (**Figure 6C**), suggesting that the source for systemic IFNγ detectable in the sera of WT mice can be NK and NKT cells, which are absent in both *Il15ra-/-* and *Il15-/-* mice (7). These observations suggest that early IFNγ production in the infected tissue following exposure to *L. monocytogenes* is IL-15-dependent but is IL-15Rα independent, and originates from cells other than NK and NKT cells.

### Exogenous IFNγ Partially Controls *L. monocytogenes* Infection in *Il15-/-* Mice

**Figure 7:**
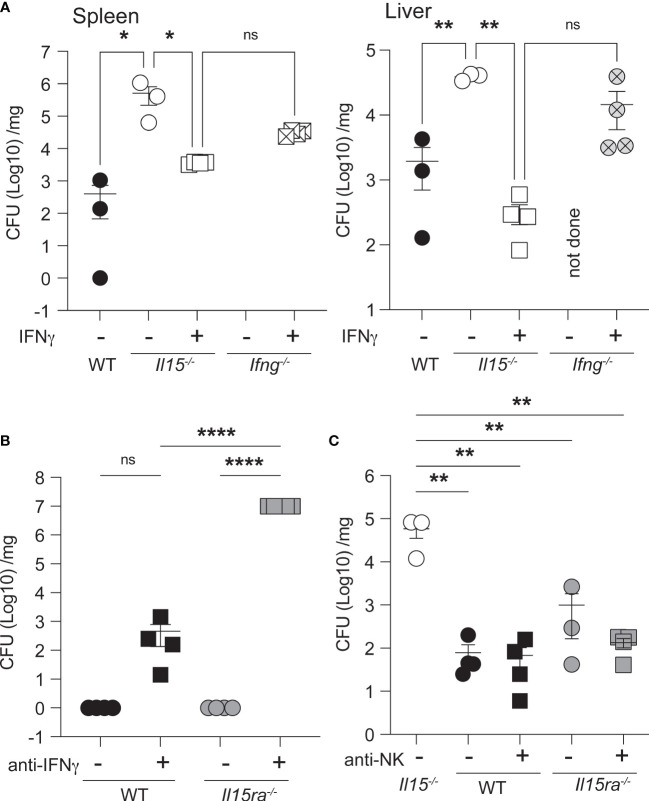
Exogenous IFNγ partially controls the bacterial load in the spleen of *Il15-/-* mice. **(A)** *Il15-/-* and *Ifng-/-* mice were infected i.v. with 5 x10^3^ CFU of *L. monocytogenes.* Infected mice received 1 x10^6^ units of IFNγ at the time of infection and at 1 dpi. Bacterial load in the spleens and livers were determined at 3 dpi. **(B)** IFNγ neutralization increases the susceptibility of *Il15ra-/-* mice. WT and *Il15ra-/-* mice received 100μg of anti-IFNγ mAb intraperitoneally on days -1 and +1 and were infected i.v. with 5 x10^3^ CFU of *L. monocytogenes* on day 0. Bacterial load in the spleen was determined at 3 dpi. **(C)** WT and *Il15ra-/-* mice received 100μg of anti-NK1.1 mAb intraperitoneally on days -1 and +1 and were infected i.v. with 5 x10^3^ CFU of *L. monocytogenes* on day 0. Bacterial load in the spleen was determined at 3 dpi. n = 3-5 mice per group from 2 different experiments. Statistical significance between groups was calculated by one-way ANOVA followed by Tukey’s multiple comparisons test. *p < 0.05, **p < 0.01, ****p < 0.0001, ns, not significant.

As IFNγ plays a crucial role in controlling bacterial infections including *L. monocytogenes* (42), we assessed whether the impaired IFNγ production in *Il15-/-* mice early during infection was the key defect underlying impaired bacterial clearance despite the elevated production of several inflammatory cytokines in the spleen. To this end, *Il15-/-* and *Ifng-/-* mice were treated with 1 x10^6^ units of IFNγ at the time of infection and at 1 dpi. IFNγ treatment reduced the bacterial load in the spleen to a modest level and almost completely in the liver of *Il15-/-* mice (**Figure 7A**), suggesting that impaired early IFNγ production plays a crucial role in the control of the infection. Exogenous IFNγ has been shown to limit *L. monocytogenes* growth in the liver more than in the spleen (43). As we had observed low levels of IFNγ in the circulation of *Il15ra-/-* mice (**Figure 6C**), we asked whether depletion of IFNγ prior to infection rendered them susceptible to low dose *L. monocytogenes*. WT and *Il15ra-/-* mice received neutralizing anti-IFNγ antibody 18 hours before infection and at 1 dpi and were sacrificed at 3 dpi. Depletion of the low levels of IFNγ present in *Il15ra-/-* rendered them susceptible to low dose *L. monocytogenes* and the mice had to be sacrificed 3 dpi (**Figure 7B**). In line with the observations that lymphocytes including NK cells are not absolutely essential for the early innate immune response in certain situations (44–47), depletion of NK cells in WT or *Il15ra-/-* mice did not increase their susceptibility to *L. monocytogenes* (**Figure 7C**). These observations indicate that IL-15-dependent but IL-15Rα-independent release of IFNγ from sources other than NK cells early during infection is critical for bacterial control.

### Hematopoietic and Non-Hematopoietic IL-15 Contribute to the Control of *L. monocytogenes*

**Figure 8:**
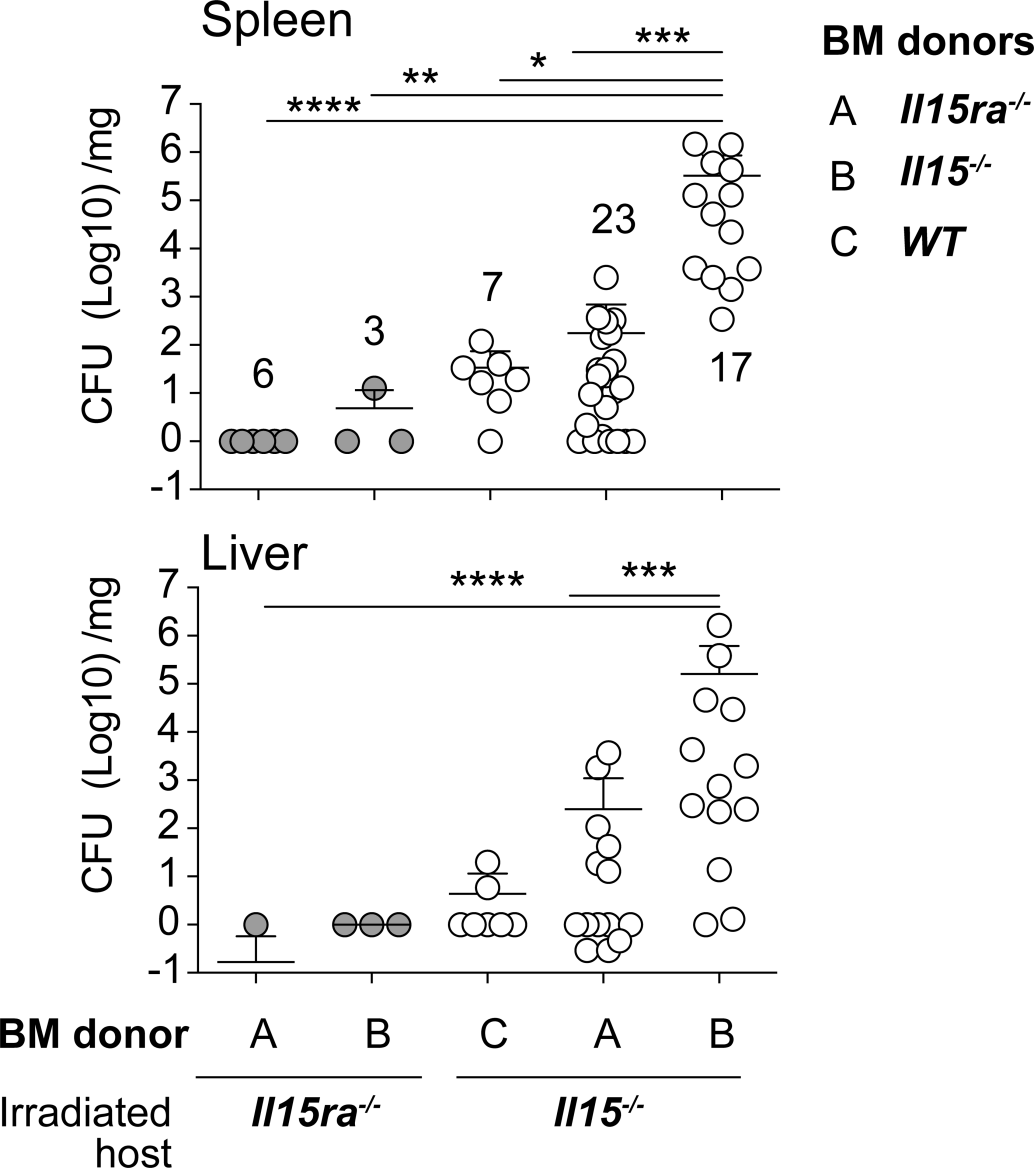
IL-15 from either hematopoietic or non-hematopoietic source is sufficient to control the infection. Two to 3 months old female WT, *Il15-/-* and *Il15ra-/-* mice received 10 Grays radiation and were reconstituted with hematopoietic cells from the bone marrow of indicated donors to generate radiation chimeras. The chimeras were infected with low dose *L. monocytogenes* two months after lethal radiation and hematopoietic reconstitution. The infected mice were sacrificed 5 dpi and the bacterial load in the spleen and liver was determined by plating on BHI-streptomycin plates. Mean ± SEM values from 2-4 independent experiments are shown. The number of recipients in each group is indicated in the figure. Statistical significance between groups, followed by Tukey’s multiple comparisons test, is given as *p < 0.05, **p < 0.01, ***p < 0.001 and ****p < 0.0001.

IL-15 from hematopoietic and non-hematopoietic cells are required for efficient activation of IL-15 dependent immune cells (7, 19, 20, 48). To determine the relative contribution of IL-15 from the hematopoietic and non-hematopoietic compartments, we generated radiation chimeras and infected them with low dose *L. monocytogenes* two months after lethal radiation and hematopoietic reconstitution. As expected, *Il15ra-/-* mice reconstituted with *Il15ra-/-* BM controlled the bacterial load, whereas *Il15-/-* mice reconstituted with *Il15-/-* BM failed to control bacterial growth (**Figure 8**). Reconstitution of *Il15-/-* mice with WT or *Il15ra-/-* BM was sufficient to control the bacterial load, and *Il15ra-/-* mice harboring *Il15-/-* BM cells still controlled the infection. These observations suggest that IL-15 originating from either the hematopoietic or the non-hematopoietic compartment is sufficient to control *L. monocytogenes* infection.

## Discussion

The finding that IL-15 is trans-presented to neighboring cells by IL-15Rα (15), the strikingly similar impact of genetically ablating IL-15 or IL-15Rα on innate and adaptive immune cell compartments (6, 24) and the dispensability of IL-15Rα on memory CD8 T cells to respond to IL-15 (49) have firmly established the concept that IL-15Rα is essential for IL-15 signaling. However, soluble IL-15 can bind the IL-15Rβγc heterodimeric receptor complex albeit with less affinity than when IL-15 is presented by IL-15Rα (15, 16). A few studies including ours have also reported that IL-15 is more critical than IL-15Rα in the context of antiviral responses, autoimmune disease and antitumor immunity (21–23). It has also been shown that IL-15Rα modulates IL-15 signaling to control the development of γδ-17 T cells and cutaneous psoriasiform inflammation (25, 50). These studies indicate that IL-15Rα-independent IL-15 signaling, presumably *via* the IL-15Rβ/γc complex is physiologically relevant for certain immune functions. The findings of the present study substantiate this notion by demonstrating that IL-15 can elicit, independently of IL-15Rα, innate immune responses following *Listeria* infection that involve early IFNγ production necessary for pathogen control by sources other than NK cells.

Even though several studies have established a role for IL-15 in conferring protection against infections (3, 14, 27, 51), few have addressed the role of IL-15Rα in pathogen control. To our knowledge, our study is the first to report the dispensability of IL-15Rα-mediated trans-presentation of IL-15 to mount the early innate responses to *Listeria*. A striking difference observed in *Listeria-*infected *Il15-/-* and *Il15ra-/-* mice was the profound decrease in the production of early IFNγ in the former but not in the latter. While the levels of IFNγ in the spleen was comparable between WT and *Il15ra-/-* mice following *Listeria* infection, WT mice displayed a 4-fold higher level of IFNγ in the serum, which is obviously derived from NK cells but seems dispensable for bacterial control. On the other hand, the induction of IFNγ within the spleen following systemic infection, which is necessary and sufficient for the activation of the early innate immune responses, was not dependent on IL-15Rα. The source of this tissue IFNγ is not yet clear. Alternate sources of the early IFNγ could include neutrophils and innate memory and H2-M3-restricted CD8+ T cells (52–57). Whereas the latter is dependent on IL-15 signaling for development (52) and thus would be unavailable in *Il15ra-/-* mice, the effect of IL-15 on IFNγ production by neutrophils has not been reported yet. IL-15 signaling has also been shown to elicit IFNγ expression in DCs (58). IL-15 expression in DCs is upregulated immediately after *Listeria* infection (59). Following *Francisella tularensis* infection, DCs accounted for 15-50% of innate IFNγ producing cells (60). As depletion of IFNγ in infected *Il15ra-/-* mice rendered them susceptible to low-dose *Listeria* infection (**Figure 7B**), it is possible that the DCs are a potential source of early IFNγ in the spleens of *Il15ra-/-* mice. Clearly, further studies are needed to identify the cell type involved in IL-15-dependent IL-15Rα-independent early IFNγ production following bacterial infection.

The source of IL-15 that confers protection against *Listeria* infection can be both hematopoietic and non-hematopoietic, as shown by the bone marrow radiation chimera experiments (**Figure 8**). Reconstitution of *Il15ra-/-* mice with *Il15-/-* bone marrow cells or vice versa conferred the ability to clear *Listeria* infection. Nevertheless, IL-15 from the non-hematopoietic cells appear to have an important role, as the bacterial load tended to be higher in *Il15-/-* recipients reconstituted with IL-15Rα bone marrow cells, suggesting that tissue-derived IL-15 has a significant role in pathogen control. IL-15 is expressed in diverse immune and somatic cell types (5, 61) and bone marrow chimeras has shown the distinct roles of hematopoietic and parenchymal sources of IL-15 in controlling lymphoid cell development and homeostasis (48). Such adoptive transfer studies and the use of conditional IL-15Rα deletion in tissues (19, 20) indicated that the dependence on tissue-derived IL-15 on immune functions appears to vary with the niche and the requirement of IL-15Rα-mediated trans-presentation. Whereas IL-15 from intestinal epithelial cells is required for maintaining lymphoid subsets in the intestine (62), lung-derived IL-15 may not be critical for the maintenance of CMV-specific memory T cells (63). As IL-15-mediated control of *Listeria* does not require IL-15Rα, further work is needed to determine how IL-15 is produced and made available to responder cells that express IFNγ needed for bacterial control.

Besides its role in IL-15 trans-presentation and facilitating ligand interaction with the IL-15Rβγ_c_ heterodimer (64, 65), IL-15Rα is implicated in regulating IL-15 availability and in modulating IL-15 signaling. IL-15Rα can be released from the cell surface by proteolytic enzymes, and this soluble form (sIL-15Rα) act as an antagonist of IL-15-dependent functions (66). Even though the trimeric IL-15Rαβγ_c_ complex is considered the functional IL-15 receptor, two lines of evidence indicate that IL-15 can signal *via* the IL-15Rβγ_c_ heterodimer. First, we have shown that IL-15 promotes autoimmune Type 1 diabetes (T1D) that does not rely on IL-15Rα but requires IL-15Rβ, as treatment with antibody targeting the IL-15Rβ during the early and late stages of insulitis progression prevented T1D development and the expansion of diabetogenic T cells (21, 22). Similar results were subsequently reported in other models of T1D (67–69). Secondly, two studies have shown that IL-15 signaling elicits divergent signals depending on the availability of IL-15Rα. Comparison of γδ T cells in *Il15ra-/-* and *Il15-/-* mice revealed an increased frequency of IL-17 producing γδ T cells in *Il15ra-/-* mice with respect to *Il15-/-* or WT mice (25). Mixed bone marrow chimeras revealed that this skewing towards γδ-17 cells arises from a cell-intrinsic phenomenon in IL-15Rα-deficient cells. Bouchaud et al., have shown that IL-15Rα deficiency exacerbated psoriatic lesions by increasing the frequency of IL-17 producing αβ and γδ T cells that was not observed in IL-15-deficient mice (50). Treatment with sIL-15Rα complexes decreased the inflammation *in vivo* and reduced the frequency of IL-15-induced IL-17 producing αβ and γδ T cells *in vitro*. Together these two studies indicated that IL-15Rα-dependent IL-15 signaling restrains the skewing of αβ and γδ T cells towards IL-17 producing cells, but did not address whether IL-15 signaling occurred *via* the IL-15βγ_c_ heterodimer. As IL-15Rα can bind IL-15 with higher affinity than that of the IL-15βγc heterodimer, sIL-15Rα may decrease the bioactive IL-15 available to promote inflammatory responses. In support, neutralization of IL-15 decreased psoriatic lesions in mice and in xenografts (70). On the other hand, exogenous IL-15 upregulates Th-17 responses of T cells in psoriasis (71), possibly by saturating the IL-15Rα containing trimeric receptor complexes and engaging IL-15βγc dimeric receptors. Collectively, in line with these studies, our current findings support the idea that IL-15 can elicit productive signaling *via* the IL-15βγc dimeric receptors, although it remains to be elucidated whether and how the downstream signaling pathways differ from those of the trimeric receptor complex.

The ability of IL-15 that is trans-presented by IL-15Rα to activate NK cells and CD8+ T cells has generated a lot of interest in exploiting it for cancer immunotherapy (72, 73). However excessive activation of NK cells and CD8+ T cells and the associated toxicity following the administration of free IL-15 has dampened its widespread application in the clinics (74). Nonetheless, the use of IL-15 conjugates in conjunction with other therapies have renewed the interest in IL-15 (75). While our knowledge on the mechanisms by which IL-15 activates lymphocytes is extensive, it is not clear how IL-15 signals in macrophages, DC and related cell types. The dispensability of IL-15Rα for eliciting such functions, as shown in our study, should overcome the limitations of trans-presentation-dependent lymphoid cell activation. Besides, dissecting the IL-15 signaling mechanisms *via* the IL-15βγc heterodimer could identify signaling pathways that could be exploited to achieve the same goals.

## Data Availability Statement

The raw data supporting the conclusions of this article will be made available by the authors, without undue reservation.

## Ethics Statement

The animal study was reviewed and approved by Université de Sherbrooke Ethics Committee for Animal Care and Use.

## Author Contributions

MN: Flow cytometry planning, experimentation and analyses, manuscript review and editing. MMM: *In vitro* infections and analyses, manuscript review and editing; SO: Initial experimentation. JMFM, AAC, GCM, and SO: infections and bacterial counts. FR and M-AL: experimentation for flow cytometry, bacterial counts. MM: generation and maintenance of mice, preparation of tissue lysates, gene expression analyses. SI: Hypothesis generation, manuscript reviewing and editing. AM and SR, Hypothesis generation, Conceptualization, Securing funding, Supervision, Data analysis and interpretation, Manuscript- writing, reviewing and editing. All authors are in agreement with the submitted version. All authors did indeed contribute.

## Funding

This work was supported by NSERC Discovery grants to SR (RGPIN-2016-04349) and AM (RGPIN-2018-05404).

## Conflict of Interest

The authors declare that the research was conducted in the absence of any commercial or financial relationships that could be construed as a potential conflict of interest.

## Publisher’s Note

All claims expressed in this article are solely those of the authors and do not necessarily represent those of their affiliated organizations, or those of the publisher, the editors and the reviewers. Any product that may be evaluated in this article, or claim that may be made by its manufacturer, is not guaranteed or endorsed by the publisher.
