# Supplementary material for: IL-15Rα-Independent IL-15 Signaling in Non-NK Cell-Derived IFNγ Driven Control of Listeria monocytogenes
Source: Front Immunol. 2021 Dec 10;12:793918. doi: 10.3389/fimmu.2021.793918 (PMC8703170; doi:10.3389/fimmu.2021.793918)
Supplement: Supplementary file 1 [file DataSheet_1.pdf]

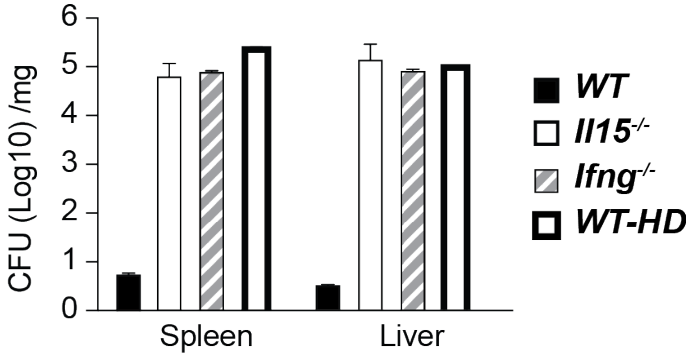

Supplementary Figure S1.

Mice lacking IL-15 or IFNγ infected with a low dose inoculum reach the same bacterial loads as WT mice infected with high dose *L. monocytogenes*.

WT, *Il15*$^{-/-}$ and *Ifng*$^{-/-}$ mice were infected i.v. with a low dose (5 x10^3 CFU) of *L. monocytogenes* and WT female mice infected with a higher dose (50-100 x10^3 CFU) of *L. monocytogenes* (WT-HD). The infected mice were sacrificed at 3 dpi and the bacterial load in the spleen and liver was determined. N=4 per group.

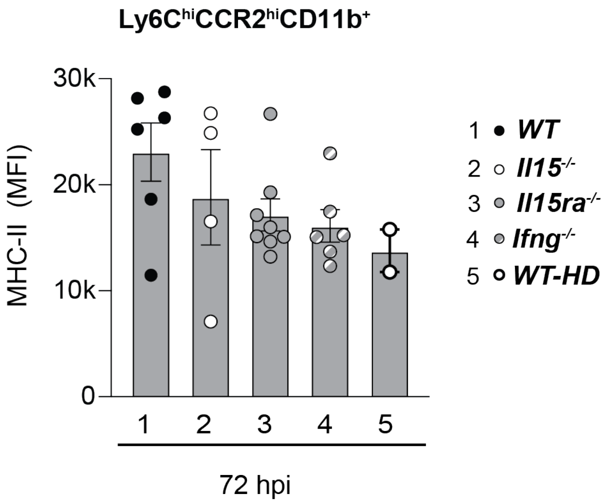

Supplementary Figure S2.

MHC Class II expression on inflammatory monocytes in *Il15*$^{-/-}$ mice following *L. monocytogenes* infection.

Mean fluorescence intensity (MFI) of MHC class-II expression in Ly6C^{+}CCR2^{+} inflammatory monocytes described in Figure 5. No statistical significance was observed between the groups.

Supplementary Table 1

List of antibodies used in this study

MARKER	FLUOROCHROME	STAINING	CLONE	CATALOG NO.	SOURCE
CD11b	eFluor450	Surface	M1/70	48-0112-82	Invitrogen
CD11c	PE-Cy7	Surface	HL3	558079	BD Biosciences
Ly6C	APC-Cy7	Surface	AL-21	560596	BD Biosciences
Ly6G	PerCP	Surface	1A8	127654	BioLegend
MHC-I	APC	Surface	AF6-88.5.5.3	17-5958-82	eBioscience
MHC-II	V500	Surface	M5/114.15.2	562366	BD Biosciences
F4-80	Brilliant Violet 605	Surface	BM8	123133	BioLegend
CCR2	PE	Surface	SA203G11	150610	BioLegend
CX3CR1	PE/Dazzle 594	Surface	SA011F11	149014	BioLegend
iNOS	APC	Intracellular	CXNFT	17-5920-82	eBioscience
